# Understanding the Relationship Between Subjective Wellbeing and Gambling Behavior

**DOI:** 10.1007/s10899-017-9692-4

**Published:** 2017-04-26

**Authors:** Lisa Farrell

**Affiliations:** School of Economics, Finance and Marketing, RMIT University, Melbourne, VIC 3000 Australia

**Keywords:** Gambling, Happiness, DSM-IV, PGSI, Subjective wellbeing

## Abstract

This paper examines the relationship between gambling behavior and subjective wellbeing. It is often asserted that populations consist of different types of gamblers: those for whom gambling is a harmless leisure activity and those (pathological/problem gamblers) for whom the activity has harmful effects. One might, therefore, assume that subjective wellbeing will be negativity associated with an individual’s level of gambling addiction. Alternatively, gamblers may choose to gamble because they derive utility from participating in this activity and so the relationship between happiness and gambling might be positively correlated. In this paper we test this association, empirically, using data from the 2010 British Gambling Prevalence Survey. The statistically significant findings from this analysis support the hypothesis that individual wellbeing falls as gambling disorder increases.

## Introduction and Background

In the context of rapidly expanding betting and gambling markets, it is important to understand the relationship between gambling behaviors and wellbeing. The harm relating to excessive gambling has been well documented in terms of both the private costs (to individuals) and social costs (to society). The disruption caused by compulsive gambling to individual welfare was recognized in 1980 when the American Psychiatric Association formally acknowledged pathological gambling as a defined ‘Disorder of Impulse Control’, setting out criteria in its *Diagnostic and Statistical Manual III* (DSM-III) (American Psychiatric Association 1980). Since then, a number of updates to the DSM-III screen have occurred, alongside a number of variations to this inventory. Psychometrically sound gambling disorder screens incorporate detailed behavioral traits. The multidimensionality of clinically based screens is illustrated by the fact that they typically cover criteria such as being preoccupied with gambling, the need to gamble increasing amounts of money, chasing losses, irritability when not gambling, escapism, denial of behavior to family, committing crime to support the activity, disruption to relationships and financial stress. Evidence suggests that acute pathological gambling is one of the most difficult disorders to treat (Volberg 1996). Hence, it is probable that gambling behaviors will impact negatively on an individual’s wellbeing. Moreover, it is possible to measure subjective wellbeing through the utilization of general/overall happiness scales. This paper will empirically investigate the statistical association between gambling behavior and wellbeing through the utilization of a happiness scale and two measures of gambling behavior (i) the DSM-IV gambling disorder inventory and (ii) the problem gambling severity index (PGSI).

Gambling participation poses an interesting conundrum for behavioral researchers. Given that the expected return from a gamble is negative, rational economic agents ought to choose not to participate. Gambling is economically illogical; yet people gamble. A common solution offered to this irrationality problem is to posit a utility-enhancing, non-pecuniary component to gambling participation, which ensures that the net effect of gambling participation on utility is positive and therefore welfare enhancing. Economists refer to this as the expected ‘utility plus *fun* framework’.^1^ The dream function literature suggests that gambling is wellbeing/happiness enhancing through the selling of hope, the buying of a dream, and the like, thus facilitating escapism (see, for example, Conlisk 1993; Simon 1998). Consistent with this behavioral decision-making approach to understanding gambling participation, the gambling industry keenly portrays gambling as a happiness-generating (utility-giving) leisure activity. Gambling advertising focuses on the thrill of the win and the life-changing aspects of big wins. Images of smiling people at casino tables or gaming machines are common. But given the potential for harm, is it really the case that gambling is wellbeing enhancing? Are gamblers happier than people who abstain from gambling? In the expected utility plus fun framework, individuals with a taste or preference for gambling activities will continue to participate up to the point at which they no longer derive positive utility from this activity, that is, when the *fun* component of the gambling activity (the positive psychological effects) is insufficient to compensate for the negative expected return of the gamble. At that point we would expect to see them switch (substitute) to alternative leisure activities. However, given that gambling is potentially addictive and defined as an impulse control disorder, whereby gambling becomes a compulsive activity, it is probable that addicted individuals may not be able to transfer their consumption to other activities/products if the impulse to gamble is sufficiently strong. Hence, they may continue to participate in gambling activities beyond the point at which their participation stops generating positive utility. In other words, those with a gambling disorder will be unable to control the impulse to gamble despite the irrationality of this behavior from a wellbeing perspective (which is inconsistent with the traditional economic utility maximization theory of consumption).^2^ Incorporating addiction affects suggests that they may gamble past the point at which subjective wellbeing declines, suggesting an inverse relationship between gambling disorder and subjective wellbeing.

From an applied research perspective, a useful development in the science of quantifying wellbeing has been the focus on subjective wellbeing. Here we concentrate on the measure of subjective wellbeing that relates to an individual’s overall happiness with his/her life—that is, a global, context-free, definition of life satisfaction. The literature developed over the past two decades, in particular, has established statistical consistencies that validate the use of ‘happiness with life’ scales as a robust measure of wellbeing (see, among others, Diener 1994). The literature on the socioeconomic determinants of ‘happiness with life’ is also well developed (for a review, see Dolan et al. 2008). Subjective wellbeing as a measure of quality of life is a psychological standard, yet much of the literature has focused on the depression and other negative psychologies. The rational for this is that by reducing negative emotions we can increase quality of life. Empirical research generally supports the notion of a significant association between gambling and depression. For instance pioneering studies (see, e.g., Blaszczynski et al. 1990; McCormick et al. 1984; Törne and Konstanty 1992) provide evidence of a positive association between gambling and depression. More recent studies include (Blanco et al. 2012; Moghaddam et al. 2015; Quigley et al. 2015; Savron et al. 2001) among others. However, mental health is a complex mixture of both negative and positive emotions. Studying the relationship between gambling and positive psychology allows us a direct way to understand the impact on positive feelings of happiness and life satisfaction. To understand the full the impact of gambling on mental health we need to examine the impact on both the negative and positive psychology of gamblers. Hence, understanding the relationship between subjective wellbeing and gambling behaviors is just as important as understanding the relationship with depression. Moreover, while negative and positive emotions are likely to be correlated it is not the case that there is prefect correlation, that is, it is not the case that happiness is the inverse of depression. For example, see Zheng (2016) for a study of the correlation between subjective wellbeing and depression.

In relation to gambling behaviors, there are a few studies that have empirically investigated the proposition that gambling generates happiness; but most of the literature is focused on a particular form of gambling and often the samples only contain gamblers. Results showing positive effects of recreational gambling have been reported in studies of the elderly. For example, Vander Bilt et al. (2004) recorded emotional responses (such as, smiling) while individuals were engaged in the act of simulated gambling on a laptop, offered a choice between playing slot machines, standard video poker, roulette, blackjack or craps. Studies of this nature focus on mood enhancement, arousal and excitement generated by gambling participation but have less to say in terms of the longer-terms effects on overall happiness. Dixon et al. (2010), in a study that also considered the elderly, found that participation in bingo, outside the home, was positively associated with happiness. However, the discussion of their results focused on social support as the explanation for this observation (given the known issues around social isolation facing this particular age group), as opposed to the consumption value (the ‘fun’) of the gambling activity.

Studies that employ a gambling inventory to capture gambling behaviors include that of Ohtsuka et al. (1997), who showed that happiness is inversely related to scores on the South Oaks Problem Gambling Screen (SOPGS) among a sample of gaming machine (pokies) players in Australia. The SOPGS is a gambling disorder screen designed to discriminate between different sub-clinical levels of gambling behaviors. The study surveyed gaming machine gamblers upon exit from a gaming venue. Respondents were surveyed immediately after participating in the act of gambling so it is unclear whether mood enhancement or impacts on wellbeing were being captured. Furthermore, given that this was a sample of gamblers only, for a particular form of gambling, it is hard to generalize the results.^3^ In a Japanese population-based sample, Shiue (2015) found that self-reported gambling addicts were most likely to report fair to poor self-rated health and fair to poor self-rated happiness relative to the rest of the population, but the survey instrument used to capture gambling disorder in these data was based on self-reporting of addiction, which is likely to under-record the number of addicts, relative to a screening instrument, given that denial is a common factor associated with a gambling disorder.

At the extreme, suicidal ideation, suicide attempts and suicides themselves can be considered clear indicators of a lack of happiness or satisfaction with one’s life and there is a body of literature that examines the relationship between suicide, suicidal ideation and suicide attempts in relation to gambling behaviors. For example, Petry and Kiluk (2002) conducted a study of pathological gamblers which revealed high suicide rates; and Newman and Thompson (2003) undertook a population-based study in which mental illness was found to be a mediating factor. From a policy perspective, Phillips et al. (1997) suggest that legalized gambling leads to elevated suicide levels in local populations and among visitors to locations associated with high levels of gambling activities. However, given that suicide is somewhat different from subjective wellbeing (although clearly associated), we recognize this body of literature but do not discuss it further.

This paper contributes to the extant literature by investigating the relationship between gambling behaviors and happiness, in a large, population-based sample. This allows us to understand the longer-term effects on overall general happiness with life, rather than the immediate impact on mood. Where individuals are not being observed in the act of gambling, it is necessary to utilize a screening instrument to capture the degree of addiction/attachment to gambling activities. Here we employ the DSM-IV gambling disorder inventory, and the PGSI and we know of no other published work that relates either the DSM-IV or PGSI directly to general happiness. This dearth in the literature may in part be due to the paucity of data that contains information on both subjective wellbeing and gambling addiction measures. Population-based surveys often ask respondents about their frequency of participation, duration of participation or gambling expenditure, and these variables have been used to measure gambling behaviors. However, Walker (1989) suggests that if the central motivation of gamblers is the desire to win money rather than the pleasure intrinsic to the activity itself, then simply losing too much money falls short of the criteria necessary to define an addictive state. This raises an important point: attachment to the gambling market is a multifaceted concept, the principle components, or domains of behavior, of which are captured most accurately by gambling disorder screens. Gambling behavior (measured according to dependency or possible harm) can be summarized by an individual’s score on a gambling disorder screen. A low score represents low levels of dependency on gambling and a high score represents levels of gambling dependency that lead to disruption and harm in the individual’s life. Population-based studies have the added advantage of allowing us to observe the full range of gambling behaviors, from abstainers through to pathological gamblers. Here we consider both the DSM-IV gambling disorder inventory (which has been designed primarily to identify clinically diagnosed gamblers from other types of gamblers and abstainers) and the PGSI (which is designed specifically for population based surveys, to discriminate between different types of gambling behaviours at both the clinical and sub-clinical levels of addiction).

## Data Description and Research Design

The data employed for this investigation is taken from the British Gambling Prevalence Survey 2010 (BGPS). This is the third large-scale, nationally representative survey of gambling participation and problem gambling prevalence in Great Britain. The 2010 survey was the last in this series of datasets collected by the United Kingdom Gambling Commission and so represents the most current data available from which to conduct our analysis.^4^ The data is ideal for our purposes in that it records gambling addiction scores across the population and asks individuals a selection of questions relating to their health and wellbeing. Specifically, it asks individuals about their general happiness levels. This data is unique in that we know of no other large population-based datasets that contain measures of both subjective wellbeing and gambling behaviors. The data was collected by the National Centre for Social Research via computer assisted self-interview. Respondents are aged 16 and over. Ethics approval for the survey was given by the National Centre for Social Research’s independent ethics review panel. The total sample size is 7756 people but item-level non-responses resulted in an estimation sample of 6624 people. No further data trimming has been conducted as the majority of the data is categorical in nature so observing outliers is not possible and we have no way to observe individual level measurement error. The estimation sample descriptive statistics are presented in Table 3.

### Dependent Variable: Happiness

**Fig. 1:**
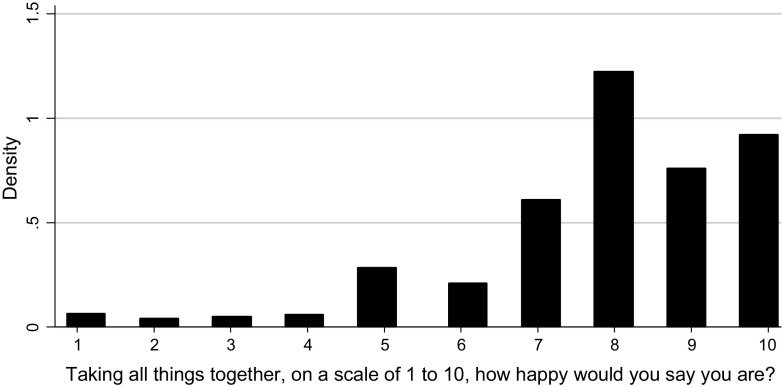
Happiness distribution

The subjective wellbeing question contained in the dataset asks respondents, ‘Taking all things together, on a scale of 1–10, how happy would you say you are these days?’ The distribution of responses is shown in Fig. 1 and has the known rightward skew with a modal value of 8. The mean value is 7.89, with a standard deviation of 1.90.

### Key Covariate: Gambling Disorder

Gambling disorders in these data are recorded via two survey instruments the DMS-IV and the PGSI. The version of the DSM-IV instrument (see Table 4; American Psychiatric Association 1994) is a 10-item scale with question topics (diagnostic criteria) ranging from chasing losses to committing crime for the purpose of funding gambling activities. Each item is assessed on a four-point scale. The item response is then dichotomized such that a ‘negative’ response is coded as 0 and a ‘positive’ response is coded as 1.^5^ The total number of positive responses is then summed to generate the respondent’s DSM-IV score (ranging from 0 to 10).^6^ Importantly for this study, the data calculates every respondent’s DSM-IV score. Non-gamblers are recorded as having a 0 score and many current gamblers also score 0. It is noteworthy that the survey defines non-gamblers as those who have not gambled in the last 12 months. This group will contain individuals who have never gambled as well as low-frequency gamblers and past (ex) gamblers. This group is therefore best defined as those who have abstained from gambling for a period of at least 12 months. However, for simplicity, we shall simply refer to this group as abstainers. A DSM-IV score of 0 and having not gambled in the last 12 months identifies ‘abstainers’. For those who have gambled in the last 12 months, a score of 0, 1 or 2 identifies ‘social’ gamblers; a score of 3 or 4 identifies ‘at risk’ gamblers; and a score of 5 or above identifies ‘pathological’ gamblers. Given that the DSM-IV gambling screen is intended for the clinical diagnosis of pathological gamblers, it defines only a single threshold for pathological gamblers. However, the use of sub-clinical thresholds (such as those defined above) has become common practice as the instrument has been more broadly utilized in population-based samples. Sub-clinical forms of the disorder can pose significant disruptions to an individual’s life and therefore represent gambling-related problems. Indeed, a deeper understanding of gambling disorder can be obtained by considering the full spectrum of gambling behaviors, including that of abstainers, social gamblers, at risk gamblers and pathological gamblers. Statistical analysis is usually conducted at these definitional levels but the underlying score is an integer ranging from 0 to 10 (an 11-point scale), representing an increasing level of the severity of harm/disruption to life resulting from the person’s gambling behaviors. This suggests that gambling behaviors are in fact a continuum, with non-problem gambling at one end and extreme pathological gambling at the other, with a range of increasingly disruptive/harmful behaviors in between. However, it is not necessarily the case that all gamblers will move up the scale and develop a clinically diagnosed gambling disorder. Our modeling approach will investigate both the discrete changes associated with the definition-based approach and the continuous underlying latent propensity to gamble approach. We wish to understand whether there is information in the thresholds themselves or whether they simply offer a convenient typology with which to classify differential gambling behaviors within and across populations.

There has been some concern about the applicability of clinical-based DSM-IV screens to population-based samples. Clinicians diagnose pathological gambling when a person meets five out of the ten criteria. In the population a wide range of gambling behaviors exist, but the intended use of clinical screens is to identify excessive behaviors and so these screens may not be useful as a measure of the full spectrum of gambling behaviors. In light of this criticism, alternative instruments have been developed such as the Population Gambling Severity Index (PGSI). This index is composed of nine items taken from the longer Canadian Problem Gambling Inventory (CPGI) (Ferris and Wynne 2001) and focuses on the harms and consequences associated with problem gambling (see Table 5). Each item is assessed on a 4 point scale and scored accordingly: never = zero; sometimes = one; most of the time = two; almost always = three. The scores for each question are then summed and a final score for each respondent ranging from zero to 27 is obtained. A PGSI score of zero is categorised as a non-problem gambler; a score of 1–2 identifies a low risk gambler; 3–7 identifies a moderate risk gambler and a score of 8 plus identifies a problem gambler. It is important to note that while the top categories in both scales equate to individuals who lives are being heavily impacted by gambling it is not the case that they discriminate at the same thresholds of gambling behaviour. The DSM-IV is identifies pathological gamblers whereas the PGSI identifies problem gamblers. Problem gamblers represent a broader concept than pathological gamblers and consistent with this idea in our estimation sample we observe 30 pathological gamblers compared to 49 problem gamblers. The correlation coefficient between the DSM-IV scale and the PSGI scale (for our estimation sample) is 0.7523 (*p* value 0.000). It is expected that this correlation will be high, but the fact that the two instruments are not perfectly correlated justifies conducting our analysis for both these measures of addiction.

We therefore conduct our analysis using both the DSM-IV and PGSI instruments. Testing the scale validity for our estimation sample, we obtain a respectable Cronbach’s alpha of 0.82 for the DSM-IV scale and 0.90 for the PGSI. The sample data shows that approximately 75% of the British population have gambled in some form over the last 12 months. Pathological gamblers combined make up 0.5% of the total population, while problem gamblers make up just less than 0.7%. This figure is externally validated by the United States data which shows that severe gambling problems are experienced by about 1% of the total population (see Kessler et al. 2008 and Shaffer et al. 1999, among others).

### Descriptive Analyses

**Fig. 2:**
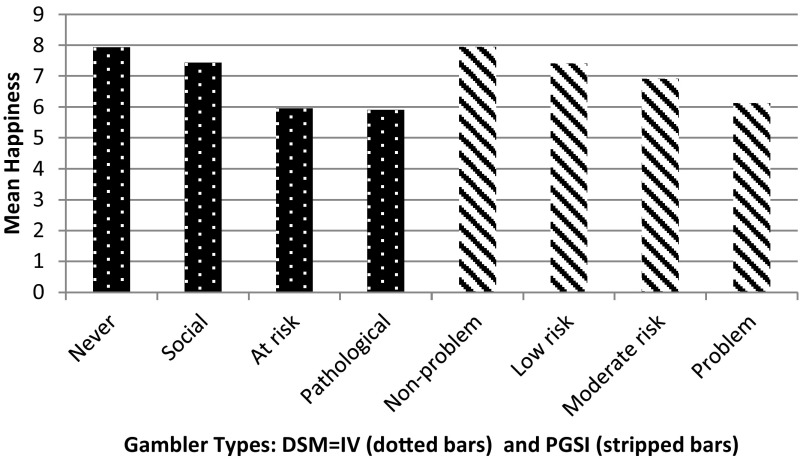
Mean happiness by gambler type

In order to consider the correlations in our data between happiness and gambling disorder scores we look at the mean (average) level of happiness for each type of gambler (DSM-IV: abstainer, social gambler, at risk gambler and pathological gambler and PGSI: non-problem, low risk, moderate risk and problem gambler). As already noted allowing for missing observations our estimation sample contains 6624 observations and the descriptive analysis is presented for this sample. The raw data suggests that there is a negative correlation between gambling disorder and general happiness, evidenced by the simple correlation co-efficient for DSM-IV and happiness of −0.114 (*p* value 0.000) and −0.1166 (*p* value 0.000) for happiness and the PGSI screen. Figure 2 illustrates the mean happiness levels by gambler type for both addiction instruments (DSM-IV and PGSI) and suggests similarities in levels of happiness between abstainers and social gamblers, and between at risk and pathological gamblers, from the DSM-IV screen. The biggest fall in happiness, of 22%, occurs when individuals move from social gambling to being at risk of developing a gambling disorder. The PGSI also shows a decline in happiness as gambling problems increase. What is interesting is that PGSI shows a more consistent fall as we move up the gambling intensity categories when compared to the stepped function we see from the DSM-IV gambler categories. This may be a result of the fact being that the DSM-IV is designed to identify clinical populations, whereas the PGSI is designed to discriminate sub-clinical thresholds of behaviour. In order to explore these findings further, we present our multivariate analysis.

### Modeling Approach

The accepted approach to empirically modeling happiness scales is to use an ordered probit model as the outcome variable takes an ordered integer value from 1 to 10 (Greene 2000).^7^ Here we adopt this methodology and employ robust standard errors (White 1984).

Our estimation equation is: $H_{i} = \alpha + \beta_{1} X_{i} + \beta_{2} GB_{i} + \varepsilon_{i}$, where $\varepsilon_{i} \sim N\left( {0,\sigma } \right),$ where GB is the individual’s gambling behaviors which are captured in our analysis by level of gambling disorder, measured via the DSM-IV or PGSI instrument.

In our analysis, levels of gambling disorder are defined in two ways. In our first specification we include a set of dummy variables representing the types of gambler (For DSM-IV: abstainer, social, at risk or pathological and for PGSI: non-problem, low, moderate or problem gambler). In our alternative specification we include the individual’s score on the DSM-IV or PGSI gambling disorder screen and treat it as a continuous variable. It should be noted that the issue of cell size is important for the gambling type analysis. While the cell sizes are representative of the relative frequencies of the different types of gamblers in the population, it is nevertheless important to consider the impact from a statistical perspective. With small cell sizes in an explanatory categorical variable the ordered probit results may be unstable or simply not converge. This is not the case here and the models converge in three iterations and the results are very stable to small changes to the model specification. The usual applied solution to small cell sizes is to reduce the number of categories by merging categories together to form larger cells. In the case of addiction screens that are designed to identify different behaviors it is not clear if merging categories together is either appropriate or informative and for this reason we present the results for the full range of gambler types, but we note that small sizes in the case of pathological (DSM-IV) and problem (PGSI) gamblers suggests these coefficient estimates should be treated with caution.^8^ As a further robustness test of the gambler type results is provided by the continuous variable for gambling addiction analysis. In this alternative specification we treat the DSM-IV/PGSI score as a continuous variable. In this functional form we are no longer thinking of gamblers as discreet types, instead addiction is being thought of as a continuous spectrum and so the issue of small cell sizes is no longer relevant: it has been assumed away in the assumption that gambling addiction can be represented as a continuous spectrum rather than as discreet types of gamblers.

All model specifications also include a vector of individual-level demographics and socioeconomic characteristics *X*_*i*_, as control variables. These characteristics are gender, age, highest educational attainment, marital status, ethnicity, family structure (the number of adults and children in the household), current employment status and personal income (reported in 12 bands). We also include in this vector a variable for general heath (‘very good/good’, ‘fair’, ‘bad/very bad’), as it is well known that poor health impacts on subjective wellbeing.^9^ Finally we controlled for country level differences across England, Scotland and Wales. As gambling legislation does not differ across Britain opportunities to gamble are not regionally determined, so we would not expect to see supply side effects, although there may be current or historic licencing effects. However, there could be demand side effects arising from cultural differences in the acceptance of gambling behaviours across England, Scotland and Wales. If present these licencing and cultural effects will be captured in the country level dummy variables. The full set of results is shown in Table 1.^10^

**Table 1:** Ordered probit estimation results

Number of observations = 6624	DSM-IV: gambler type		DSM-IV		PGSI: gambler type		PGSI	
	Coef	P > |Z|	Coef	P > |Z|	Coef	P > |Z|	Coef	P > |Z|
Demographics								
Female	0.099	0.000	0.100	0.000	0.085	0.002	0.094	0.001
Age	−0.014	0.010	−0.014	0.009	−0.015	0.006	−0.014	0.010
Age squared	0.000	0.000	0.000	0.000	0.000	0.000	0.000	0.000
Education								
Other qualification	0.176	0.127	0.179	0.121	0.177	0.125	0.180	0.120
‘O’ levels	−0.131	0.002	−0.128	0.002	−0.128	0.002	−0.127	0.002
Professional or above	−0.125	0.003	−0.123	0.004	−0.128	0.003	−0.122	0.004
Marital status								
Separated/divorced	−0.251	0.000	−0.253	0.000	−0.252	0.000	−0.254	0.000
Single/never married	−0.213	0.000	−0.215	0.000	−0.211	0.000	−0.214	0.000
Widowed	−0.573	0.000	−0.572	0.000	−0.570	0.000	−0.574	0.000
Economic activity								
Unemployed	−0.319	0.000	−0.316	0.000	−0.316	0.000	−0.319	0.000
Long-term disability	−0.213	0.012	−0.214	0.012	−0.214	0.012	−0.221	0.009
Looking after family/home	0.038	0.497	0.039	0.488	0.038	0.499	0.036	0.521
Retired	0.187	0.001	0.187	0.001	0.185	0.001	0.184	0.001
Full-time education	0.049	0.433	0.052	0.406	0.055	0.385	0.060	0.339
Other	−0.140	0.111	−0.144	0.103	−0.150	0.089	−0.145	0.102
Ethnicity								
Asian or Asian British	−0.225	0.001	−0.220	0.002	−0.233	0.001	−0.226	0.001
Black or Black British	−0.158	0.055	−0.157	0.057	−0.148	0.073	−0.154	0.062
Other ethnic group	−0.369	0.000	−0.368	0.000	−0.354	0.000	−0.360	0.000
Household structure								
Two adults in household	0.189	0.000	0.190	0.000	0.189	0.000	0.186	0.000
Three adults in household	0.083	0.128	0.082	0.133	0.081	0.139	0.076	0.167
Four adults in household	0.203	0.001	0.202	0.001	0.201	0.001	0.198	0.001
Household with children	−0.035	0.275	−0.035	0.273	−0.036	0.264	−0.035	0.281
Income								
Personal income	0.006	0.241	0.006	0.239	0.006	0.281	0.006	0.270
Health status								
Fair health	−0.382	0.000	−0.383	0.000	−0.381	0.000	−0.382	0.000
Bad/very bad health	−0.715	0.000	−0.715	0.000	−0.710	0.000	−0.708	0.000
Region								
Wales	−0.025	0.633	−0.025	0.636	−0.023	0.667	−0.024	0.640
Scotland	0.055	0.211	0.057	0.197	0.057	0.192	0.057	0.196
Gambling addiction measure								
DSM-IV: social	−0.144	0.065						
DSM-IV: at risk	−0.757	0.000						
DSM-IV: pathological	−0.710	0.005						
DSM-IV			−0.131	0.000				
PGSI: low					−0.228	0.000		
PGSI: medium					−0.470	0.000		
PGSI: problem					−0.562	0.002		
PGSI							−0.058	0.000
Log Pseudolikelihood	−11,743.324		−11,742.914		−11,735.784		−11,741.744	

*Note* Omitted: male, no qualifications, married/living as married, in paid work, white ethnic origin, adult only household, very good/good health, living in England and column (1) abstainers, column (3) non-problem gamblers

## Results

The first thing to note from our findings is the consistency of the sign and size of the coefficients on the control variables across the all specifications. Moreover, the parameter estimates are consistent with the existing literature (see review by Dolan et al. 2008). Females are happier than males, happiness has a U-shaped age relation (a negative relationship with age and a positive relationship with age squared), being married generates happiness, non-whites are less happy than whites, and good health is important for happiness. We also find that happiness declines with educational attainment, but income is not a significant predictor of happiness. In regard to country level effects we see no statistically significant effects suggesting a high degree of assimilation across Britain.

With respect to gambling behaviors, in terms of the discrete analysis, for the DSM-IV gambler types we find no statistical difference between abstainers and social gamblers (at the 95% level of significance). However, those at risk of developing a gambling disorder and pathological gamblers (those with a clinically defined gambling disorder) show statistically lower levels of happiness than abstainers. This suggests that the influence of gambling and happiness on each other remains neutral until the point where the activity starts to produce a noticeable level of disruption in individuals’ lives. These findings are consistent with the fact that the DSM-I inventory is designed to identify clinical levels gambling disorder and the group of clinically addicted gamblers have lower levels of happiness than those whose gambling behaviours is sub-clinical. For the PGSI gambler types we find that all types of gambler (low risk, moderate risk and problem gamblers) have lower levels of happiness than non-problem gamblers and that the size of the effect increases as we move through the gamblers types in order of the severity of the gambling problem. These results are consistent with the idea that the PGSI is effective in discriminating between sub-clinical differences in gambling behaviors and these gambler types are statistically correlated with different levels of subjective wellbeing.

Next we consider the alternative specification employing the continuous DSM-IV or PGSI scores. For both instruments, we see the expected negative association between happiness with one’s life and an individual’s gambling disorder score. These results are naturally intuitive and consistent with the descriptive analysis and the discreet analysis of gambler types.

Arguably a deeper understanding is obtained from the discrete variable specification relative to the continuous variable specification. The results from the DSM-IV gambler types suggest that the thresholds for both the clinical and sub-clinical gambler types are defined at important points in relation to the degree of disruption to a person’s life and that these thresholds are reflected in levels of overall happiness. Further the results from the PGSI gambler types suggest that there are also important sub-clinical thresholds that are related to observed changes in happiness. There is therefore an important depth of understanding to be gained from modeling the step function (employing the discrete variable) alongside the linear function (employing the continuous variable).

We tested for non-linearity in the relationship between DSM-IV scores and happiness but found no statistical evidence.^11^ This is not surprising given that the continuous variable analysis suggests that the relationship is decreasing and the discrete variable analysis suggests that it is a step function with the step down (a fall in happiness) occurring at specific thresholds at which increasing disruption to an individual’s life occurs. Sample size issues mean that we cannot perform that analysis separately by gender; instead we control for gender in our models. There are an insufficient number of female pathological and problem gamblers observed in our data for any separate gender-based analysis to be statistically robust.

In order to test which model specification best fits our data, we use the Akaike Information Criteria (AIC) and the Bayesian Information Criteria (BIC) tests. The results are presented in Table 2 below. We can see that in terms of the DMS-IC instrument the continuous DSM-IV specification is preferred by both of these tests (having the minimum value). Hence, the continuous DSM-IV specification is the best fit for the data. However, when we look at the PGSI instrument the results from the AIC and BIC conflict each other, with AIC preferring the gambler type specification and BIC preferring the continuous specification. One of the difficulties of the PGSI instrument is that is a 27 point scale and problem gambling is defined as recording as score of 8 or more. As a result there are often very low numbers of observations at top end of the scale and often no observations for particular scores (even in large samples). This is less often true in the case of DSM-IV as it is records zero to ten score, so while in large samples you usually have observations at each score the number of observations at high scores can still be small. This implies that statistically modeling with the DSM-IV will be more reliable. Hence, the continuous DSM-IV specification is the best fit for the data.

**Table 2:** Akaike information criteria and bayesian information criteria

Model	Obs	ll(null)	ll(model)	df	AIC	BIC
DSM-IV: gambler type	6624	−12,133.78	−11,743.32	39	23,564.65	23,829.79
DSM-IV: score	6624	−12,133.78	−11,742.91	37	23,559.83	23,811.37
PGSI: gambler type	6624	−12,133.78	−11,735.78	39	23,549.57	23,814.71
PGSI: score	6624	−12,133.78	−11,741.74	37	23,557.49	23,809.03

Due to the difficulty of directly interpreting the coefficients from the output of a probit regression (not in any standard way, at least), in order to understand the magnitude of the effects we analyse the marginal effects of the gambling regressors—that is, how much the (conditional) probability of the outcome variable (happiness) changes when you change the value of a (DSM-IV) regressor, holding all other regressors constant at mean values. Due to statistical considerations, the marginal analysis is presented graphically for the continuous DSM-IV gambling disorder measure only. The long right sided tail on the PGSI distribution of scores means that the predictions for high scores will necessarily be based on very small cell sizes and this will impact on the reliability of the results.

Hence we chose to conduct the marginal analysis for the continuous measure of gambling disorder as this provides 11 data points (DSM-IV 0–10) for each of the 10 happiness levels. Figure 2 shows the predicted probabilities of each level of happiness as we move up the gambling disorder scale. The sample sizes are (in part) reflected in the size of the confidence intervals, which are also plotted. It is important to remember that the mean level of happiness in our data is 8. So we will discuss the figure in relation to this average happiness level.

Beginning by considering happiness levels that fall below the average, we see that those at the bottom end of the subjective wellbeing scale, where happiness equals 1, 2, 3 or 4, are more likely to be observed at higher levels of the DSM-IV gambling disorder scale. That is, the predicted probabilities are increasing as we move up the gambling disorder scale for individuals with low levels of subjective wellbeing. For levels of happiness at scores of 5 and 6 on the subjective wellbeing scale, we see that the predicted probabilities, while increasing for low levels of gambling disorder, flatten out at higher levels. The pattern of the predicted probabilities for happiness equal to 7 is n-shaped, with the highest probability being observed at a DSM-IV score of 6.

Moving on to predictions for happiness equal to 8, we see that this happiness level has the greatest probability of being observed for individuals who score 1 on the DSM-IV inventory, with the probabilities falling as the addiction score increases. Further, having a DSM-IV score of 1 and a happiness score of 8 corresponds to the highest predicted probability, across all combinations of wellbeing categories and DSM-IV scores, at a predicted probability of 0.294. Hence, a person observed to be at the population mean level of happiness (happiness = 8) has a 29% chance of having a DSM-IV score of 1, which equates to being a social gambler.^12^

Finally, considering above average levels of happiness (scores of 9 and 10), we see that the highest probability of being observed at these levels of happiness occurs when DSM-IV is 0 and the predicted probabilities fall as DSM-IV increases. Hence, as the gambling disorder score increases we are less likely to observe people with above average levels of happiness (happiness = 9 or 10).

**Fig. 3:**
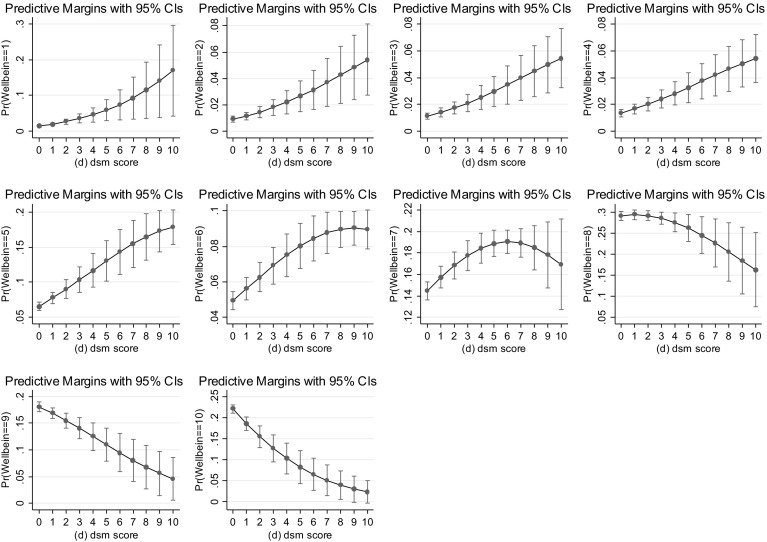
Predicted probabilities- DSM-IV continuous

In summary, the predicted probabilities illustrated in Fig. 3 show clearly that an individual’s happiness with their life is inversely related to their level of gambling addiction (as quantified via the DSM-IV gambling disorder inventory). Given that DSM-IV measures both the level of harm from gambling and the level of dependency on gambling it is not surprising to find that, as the level of disruption to life escalates, there is an observable and statistically significant fall in subjective wellbeing. The results tell us that gambling behaviors impact on overall wellbeing, as well as on the specific aspects that are contained within the 10 questions of the DSM-IV inventory relating to the social, emotional and financial aspects of gambling behavior.

## Conclusions

This paper provides evidence in support of the hypothesis that gambling addiction is negatively related to subjective wellbeing. Specifically, we identify a statistically significant negative association between general happiness and both the DSM-IV and the PGSI gambling inventories. The discrete analysis of gambler types suggests that the transition from being a social gambler to being at risk of developing a gambling disorder has important implications for general happiness. Further sub-clinical thresholds of behaviors are also associated with a fall in happiness as the intensity of the gambling problem increases. The continuous variable analysis of the DSM-IV and PGSI scores suggests that individuals with a below average level of happiness are most likely to be observed at higher scores while those at the mean or above average levels of happiness are most likely to be observed at low scores on these gambling inventories.

The findings suggest that public health policies aimed at harm minimization should focus on the transitions between different types of gamblers and better understand the pathways to pathological and problem gambling. Further, the research suggests that the impacts of gambling addiction go beyond those domains of behavior recorded in the addiction scales such as the DSM-IV and the PGSI to impacting on an individual’s holistic view of personal happiness with his/her life. This represents an important step in understanding the link between gambling addiction and more broadly defined measures of mental health such as subjective wellbeing. In particular it is important to note that these findings make a significant contribution to the existing literature which as predominantly focused on the negative psychological aspects of gambling through understanding the relationship with depression and other measures of negative thoughts. Here we have shown that there is also an impact in terms of a fall in positive thoughts from gambling behaviors by investigating the relationship with a person’s happiness. This allows us a much more holistic understanding of the impact of gambling on a person’s psychological wellbeing.

## Appendix

See Tables 3, 4 and 5.

**Table 3:** Sample characteristics: covariates

	Mean	SD		Mean	SD
Demographics			Household structure		
Gender	0.536	0.499	One household family	0.207	0.405
Age	46.642	18.025	Two adults in household	0.547	0.498
Education			Three adults in household	0.144	0.351
No qualification	0.234	0.423	Four adults in household	0.101	0.302
Other education	0.016	0.126	Household with children	0.303	0.460
‘O’ levels	0.366	0.482	Income		
Professional or above	0.384	0.486	Personal income	5.062	3.017
Marital status			Health status		
Married/living as married	0.617	0.486	Very good/good health	0.762	0.426
Separated/divorced	0.093	0.291	Fair health	0.182	0.386
Single/never married	0.230	0.421	Bad/very bad health	0.056	0.230
Widowed	0.060	0.237	Region		
Economic activity			England	0.845	0.362
Employed	0.544	0.498	Wales	0.051	0.220
Unemployed	0.030	0.172	Scotland	0.099	0.299
Long-term disability	0.035	0.184	Gambling addiction measures		
Looking after family/home	0.087	0.281	Never/not in last 12 months	0.252	0.434
Retired	0.206	0.404	Social	0.740	0.439
Full-time education	0.071	0.257	At risk	0.004	0.064
Other	0.027	0.162	Pathological	0.005	0.067
Ethnicity			DSM-IV score	0.090	0.563
White	0.914	0.280	Non-problem	0.921	0.269
Asian or Asian British	0.040	0.197	Low problem	0.056	0.230
Black or Black British	0.025	0.157	Moderate problem	0.015	0.123
Other ethnic group	0.020	0.140	Problem	0.007	0.085
			PGSI score	0.228	1.319

**Table 4:** DSM-IV diagnostic criteria used in BGPS

For the next set of questions about gambling, please indicate the extent to which each one has applied to you in the last 12 months.	
1.	When you gamble, how often do you go back the next day to win back the money you lost?
	In the last 12 months….
2.	…how often have you found yourself thinking about gambling (that is, reliving past gambling experiences, planning the next time you will play, or thinking of ways to get money to gamble)?
3.	…have you needed to gamble with more and more money to get the same excitement you are looking for?
4.	…have you felt restless or irritable when trying to cut down gambling?
5.	…have you gambled to escape from problems or when you are feeling depressed, anxious or bad about yourself?
6.	…have you lied to family, or others, to hide the extent of your gambling?
7.	…have you made unsuccessful attempts to control, cut back or stop gambling?
8.	…have you committed a crime in order to finance gambling or to pay gambling debts?
9.	…have you risked or lost an important relationship, job, educational or work opportunity because of gambling?
10.	…have you asked others to provide money to help with a desperate financial situation caused by gambling?

*Source* British Gambling Prevalence Survey 2010, Copyright© 2011, National Centre for Social Research. Appendix 3: Questionnaire documentation. (Wardle et al. 2011)

**Table 5:** PGSI Diagnostic Criteria used in BGPS

In the last 12 months, how often…	
1.	…have you bet more than you could really afford to lose?
2.	…have you needed to gamble with larger amounts of money to get the same excitement?
3.	…have you gone back to try and win back the money you’d lost?
4.	…have you borrowed money or sold anything to get money to gamble?
5.	…have you felt that you might have a problem with gambling?
6.	…have you felt that gambling has caused you any health problems, including stress and anxiety?
7.	…have people criticised your betting, or told you that you have a gambling problem, whether or not you thought that it is true?
8.	…have you felt your gambling has caused financial problems for you or your household?
9.	…have you felt guilty about the way you gamble, or what happens when you gamble?

*Source* British Gambling Prevalence Survey 2010, Copyright© 2011, National Centre for Social Research. Appendix 3: Questionnaire documentation. (Wardle et al. 2011)

## Footnotes

1 The discussion in Hartley and Farrell (2002) critiques this expected utility plus fun framework in terms of the ad hoc nature of the *fun* component.

2 The terms ‘gambling addiction’, ‘compulsive gambling’ and ‘gambling disorder’ are often used interchangeably. We recognize that the psychology literature refers to the DSM-IV inventory as a gambling disorder scale, but the broader public health and gambling studies literature frequently uses the term ‘gambling addiction’ as opposed to ‘gambling disorder’. Further, the wider public policy and advocacy group material predominantly utilizes the term ‘addiction’.

3 Unpublished work by Forrest (using the same data as this paper) is summarized in Forrest (2013), which suggests that happiness and gambling are positively associated for non-gamblers (as opposed to recreational gamblers) and negatively associated for problem gamblers. However, given that the original work is unpublished it is not possible to verify these findings.

4 Previous cross-sectional surveys were conducted in 2000 and 2007.

5 Details of the dichotomous scoring can be found in Table A2.9.2 of Wardle et al. (2011).

6 It is possible to use the non-dichotomized DSM-IV scores (ranging from 0 to 30) in the empirical analysis and the results are qualitatively the same.

7 An alternative estimator would be an ordered logit. In practice the ordered logit and ordered probit results rarely differ significantly. This is true in our case, and the results for the ordered logistic model are available from the author upon request. In the interests of comparability with the literature on subjective wellbeing, we choose to present the ordered probit results.

8 For completeness we have also rerun the analysis combining the top two categories for each addiction screen i.e. combining the at risk and the pathological gamblers in the case of the DSM-IV screen and combining the moderate gamblers with the problem gamblers in the case of the PGSI screen. The results are qualitatively the same and the papers main results are unchanged.

9 We also included indicator variables for being a smoker and/or a drinker, but these variables were not found to be statistically significant and so are not included in the final models presented here. This result may be due to collinearity between these variables and the DSM-IV scores arising from comorbidity.

10 We also tested the robustness of the findings to the inclusion of gambling expenditure, duration and frequency of participation but these variables were not found to be significant and so were dropped from the final versions of the models presented here.

11 Non-linearity was tested via the inclusion of a DSM-IV squared term. An alternative approach would be to use a log functional form but the high number of respondents recording a zero DSM-IV score means that this is not empirically sensible in this context.

12 While gamblers are categorized as abstainers and social gamblers if they record 0, 1, 2 or 3 on the DSM-IV scale, abstainers can only record a 0 by definition. Therefore, a score of 1 can only be recorded by a social gambler. Note, however, that social gamblers can also score 0.
